# Dose-escalation study of weekly irinotecan and daily carboplatin with concurrent thoracic radiotherapy for unresectable stage III non-small cell lung cancer

**DOI:** 10.1038/sj.bjc.6600464

**Published:** 2002-08-01

**Authors:** M Yamada, S Kudoh, H Fukuda, K Nakagawa, N Yamamoto, Y Nishimura, S Negoro, K Takeda, M Tanaka, M Fukuoka

**Affiliations:** First Department of Internal Medicine, Osaka City University Medical School, 1-4-3 Asahi-machi, Abeno-ku, Osaka 545-8585, Japan; Department of Radiology, Osaka City University Medical School, Osaka, Japan; Fourth Department of Internal Medicine, Kinki University School of Medicine, Osaka, Japan; Department of Radiology, Kinki University School of Medicine, Osaka, Japan; Department of Pulmonary Medicine, Osaka City General Hospital, Osaka, Japan; Department of Radiology, Osaka City General Hospital, Osaka, Japan

**Keywords:** non-small cell lung cancer, irinotecan, carboplatin, chemoradiotherapy

## Abstract

Dose-escalation study was performed to evaluate the maximum tolerated dose, recommended dose and toxicity profile of weekly irinotecan with daily carboplatin and concurrent thoracic radiotherapy in patients with locally advanced non-small-cell lung cancer. Thirty-one previously untreated patients with unresectable stage III non-small-cell lung cancer were enrolled in this study. Patients received weekly irinotecan plus carboplatin (20 mg m^−2^ daily for 5 days a week) for 4 weeks and thoracic radiotherapy (60 Gy in 30 fractions). The irinotecan dose was escalated from 30 mg m^−2^ in increments of 10 mg m^−2^. Four irinotecan dose levels were given and 30 patients were assessable. Their median age was 62 years (range: 52–72 years), 28 had a performance status of 0–1 and two had a performance status of 2, 12 had stage IIIA disease and 18 had IIIB disease. There were 19 squamous cell carcinomas, 10 adenocarcinomas, and one large cell carcinoma. The dose-limiting toxicities were pneumonitis, esophagitis, thrombocytopenia and neutropenia. The maximum tolerated dose of irinotecan was 60 mg m^−2^, with two patients developing grade 4 pulmonary toxicity and one patient died of pneumonitis (grade 5). The recommended dose of irinotecan was 50 mg m^−2^. Other grade 3 or 4 toxicities were nausea and vomiting. Three patients achieved complete remission and 15 had partial remission, for an objective response rate of 60.0%. The median survival time was 14.9 months, and the 1- and 2-year survival rates were 51.6% and 34.2%, respectively. The study concluded that the major toxicity of this regimen was pneumonitis. This therapy may be active against unresectable non-small-cell lung cancer and a phase II study is warranted.

*British Journal of Cancer* (2002) **87**, 258–263. doi:10.1038/sj.bjc.6600464 www.bjcancer.com

© 2002 Cancer Research UK

## 

In patients with unresectable stage III non-small-cell lung cancer (NSCLC), two or more cycles of cisplatin-based chemotherapy, with or followed by radiation, has been proven to enhance survival (American Society of Clinical Oncology, 1997). Chemotherapy is appropriate for selected patients who have a good performance status. In general, chemotherapy is either given first followed by radiation, or is administered concurrently with radiation. Concurrent chemoradiotherapy regimens employ chemotherapy agents as radiosensitisers. Most studies that have shown a benefit for chemoradiotherapy have used cisplatin- or carboplatin-based combinations (Dillman *et al*, 1990; Le Chevalier *et al*, 1991; Jeremic *et al*, 1995), and both drugs are known to be radiosensitizers (Schaake-Koning *et al*, 1992; Jeremic *et al*, 1996). New active agents, such as paclitaxel, docetaxel, gemcitabine, vinorelbine and irinotecan, have been introduced and clinical trials of these agents for NSCLC have yielded promising data. These agents have been compared with each other in a phase III study performed in patients with advanced NSCLC, and several studies have suggested the radiosensitising properties of these new agents (Tishler *et al*, 1992; Leonard *et al*, 1996; Mcginn *et al*, 1996; Okishio *et al*, 1996). However, the phase I and II studies combining these agents with radiotherapy have mostly been preliminary (Choy *et al*, 1994; Greco *et al*, 1996; Gregor, 1997; Mauers *et al*, 1998; Herscher *et al*, 1998). Irinotecan has a mechanism of action targeting the nuclear enzyme topoisomerase I as radiosensitiser *in vitro* (Okishio *et al*, 1996). A response rate of 32% was observed in untreated patients with advanced NSCLC (Fukuoka *et al*, 1992) while a recent phase III study showed that irinotecan in combination with cisplatin achieved a significantly better survival compared with the combination of cisplatin and vindesine in patients with metastatic NSCLC (Fukuoka *et al*, 2000). We have already reported that a phase I/II study of weekly irinotecan with concurrent radiotherapy showed acceptable toxicity (esophagitis, diarrhea, and pneumonitis)(Takeda *et al*, 1999). Carboplatin has also been investigated as a radiosensitizer. Several studies (Groen *et al*, 1995; Kunitoh *et al*, 1997; Atagi *et al*, 2000) of concurrent daily carboplatin and radiotherapy have suggested that this combination is feasible and reasonably effective. Irinotecan and carboplatin have independently shown a synergistic effect with ionizing radiation in preclinical studies (Douple *et al*, 1985; Okishio *et al*, 1996). Based on these findings, we conducted a phase I trial of daily carboplatin and weekly irinotecan with concurrent thoracic radiotherapy for the treatment of locally advanced NSCLC in order to find the optimum dose of irinotecan and to estimate the antitumor activity and toxicity profile of this therapy.

## MATERIALS AND METHODS

### Patients selection

Patients were eligible for this study if they had histologically or cytologically documented and locally advanced stage III NSCLC that was deemed unresectable. Other eligibility requirements included an age of less than 75 years, an Eastern Cooperative Oncology Group performance status (PS) of 0 to 2, no previous chemotherapy or radiotherapy, ability to give written informed consent, as well as adequate pretreatment haematologic function (leukocyte count ⩾4 000 μl^−1^, haemoglobin ⩾9.5 g dl^−1^, and platelet count ⩾100 000 μl^−1^), renal function (a normal serum creatinine concentration), hepatic function (transaminases ⩽twice the normal range and serum bilirubin level ⩽1.5 mg dl^−1^), and pulmonary function (PaO_2_ ⩾70 Torr, %DLco >60%). Patients were excluded if they had contralateral hilar lymph node metastasis, a serious pre-existing disease, or a radiation field that exceeded half of one lung. Patient's informed consent and approval of the institutional ethics committee were mandatory for participation in the trial.

### Treatment plan

Irinotecan was administered as a 90-min intravenous infusion once weekly, and carboplatin was given as a 30-min infusion (20 mg m^−2^) prior to thoracic radiotherapy daily for 5 days each week. Irinotecan and carboplatin were both administered for 4 weeks.

Thoracic radiotherapy started on day 1 and was given to a total dose of 60 Gy in 2.0 Gy fractions, which were delivered five times a week for 6 weeks using a linear accelerator (⩾4MV). The treatment volumes consisted of original and boost volumes irradiated sequentially. The initial large-field target volume consisted of the primary tumour, mediastinum, and involved hilar of supraclavicular nodes (total dose, 40 Gy), and boost dose of 20 Gy was delivered to a volume that consisted of the primary tumour and involved nodes. A combination of parallel-opposed anterior and posterior and oblique fields was used. The maximal spinal cord dose did not exceed 40 Gy. The target volume of the primary tumour included the complete extent of the visible primary tumour as defined radiographically (by computed tomography) with a minimum 1.5 cm and a maximum 2.5 cm margin around the mass.

The following therapy is optional. If the patient became operable as a result of tumour regression, surgery was done within 1 month of the completion of chemoradiotherapy. If the patient remained inoperable, two cycles of cisplatin with vindesine (cisplatin 80 mg m^−2^ day 1 and vindesine 3 mg m^−2^ on days 1, 8, 15) were given as systemic chemotherapy.

### Dose escalation schedule

The starting dose of irinotecan was 30 mg m^−2^ and this was escalated by 10 mg m^−2^ increments in every three patients. There was no interpatient escalation. The next scheduled dose of irinotecan was omitted when grade 3 leukopenia, thrombocytopenia, or grade 2 diarrhea was observed.

Both thoracic radiotherapy and intravenous carboplatin were withheld if grade 3 leukopenia, neutropenia, thrombocytopenia, or grade 4 esophagitis was observed and restarted as soon as possible after recovery to grade 3 esophagitis and grade 2 haematological toxicity.

### Dose-limiting toxicity

Dose-limiting toxicity was defined as grade 3 or 4 nonhaematologic toxicity, excluding nausea, vomiting, and alopecia, as neutropenic fever (grade 3 neutropenia and >38°C) or as grade 4 haematologic toxicity according to the WHO criteria (World Health Organization, 1979). If irinotecan was omitted two times or more due to any toxicity or radiotherapy and daily carboplatin was postponed for more than one week because of grade 3 haematological toxicity or grade 4 oesophagitis, we decided this was dose-limiting toxicity. If dose-limiting toxicity was observed in one or two out of three patients, an additional three patients were scheduled to be treated at the same dose level, and dose escalation could then continue if the toxicity was only observed in one or two out of six patients. If the dose-limiting toxicity was observed in all three patients or in more than three out of six patients, that dose was defined as the maximum tolerated dose. Recommended dose was defined the previous dose level.

### Response and toxicity evaluation

Responses were evaluated according to the World Health Organization (WHO) criteria and toxicity was assessed prior to any further non-protocol therapy according to the WHO criteria (World Health Organization, 1979). Pulmonary toxicity was recorded as Grade 0–5 according to late Radiation Therapy Oncology Group (RTOG) criteria (Robert *et al*, 1999) as follows: 0, none; 1, asymptomatic or mild symptoms, slight radiographic appearances; 2, moderate symptomatic fibrosis or pneumonitis, low-grade fever, patchy radiographic appearances; 3, severe symptomatic fibrosis or pneumonitis, dense radiographic changes; 4, severe respiratory insufficiency, continuous oxygen, assisted ventilation; and 5, fatal. All reported responses and toxicities were confirmed by independent extramural review. Survival was measured from the initiation of chemoradiotherapy to death, and survival curves were estimated using the Kaplan–Meier method (Kaplan and Meier, 1958).

## RESULTS

### Patient characteristics

Between May 1996 and July 1998, 31 patients with histologically or cytologically confirmed stage III NSCLC were enrolled in this dose escalation study. Their clinical characteristics are summarised in Table 1

**Table 1:**
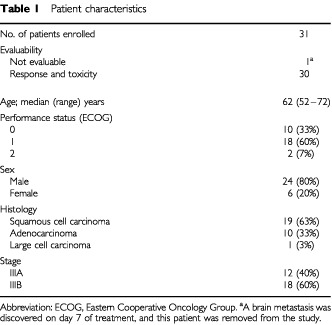
Patient characteristics

. Four dose levels of irinotecan were administered (Table 2

**Table 2:**
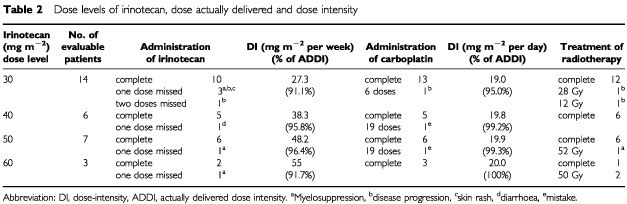
Dose levels of irinotecan, dose actually delivered and dose intensity

), and 30 patients were assessable for toxicity and efficacy. The remaining one patient who enrolled into irinotecan dose level of 50 mg m^−2^ was ineligible because brain metastasis was confirmed after enrollment. For these 30 patients, the median age was 62 years (range: 52–72 years). The performance status was 0–1 in 28 patients, while it was 2 in two patients. Twelve patients were in stage IIIA and 18 were in stage IIIB. Their tumours included 19 squamous cell carcinomas, 10 adenocarcinomas, and one large cell carcinoma.

### Actual doses of chemotherapy and radiotherapy

The planned individual drug doses, the actual delivered doses and dose intensity are listed in Table 2. Fourteen patients were treated with 30 mg m^−2^ of irinotecan. Although six patients should have been the maximum number in one step in our protocol, we added eight patients in first step to carry out this protocol safely because grade 4 pulmonary toxicity was observed in one patient, in the former study (Takeda *et al*, 1999) of weekly irinotecan combined with concurrent thoracic radiation therapy we experienced the treatment related death of pneumonitis and the Monitoring Committee of this protocol decided to add more patients in initial step. Administration of irinotecan was withheld due to neutropenia in three patients, disease progression in two patients, and diarrhea and localized erythema in one patient. Three patients did not complete the intravenous carboplatin schedule, one due to disease progression and the other due to a mistake about administration times. Dose intensities of irinotecan and carboplatin are listed in Table 2. The percentage of actually delivered dose-intensity of irinotecan and carboplatine was range from 91.1% to 100%. Twenty-five out of the 30 patients (83.3%) completed their radiotherapy as scheduled. The reason for not completing radiotherapy was disease progression in two patients and thrombocytopenia in one patient. Also, the first patient who received 60 mg m^−2^ of irinotecan suffered treatment-related death from pneumonitis and thrombocytopenia, so the other two patients treated at this dose level discontinued radiotherapy after 50 Gy.

### Haematologic toxicity

Thirty patients were assessable for haematologic toxicity, and the results summarised in Table 3

**Table 3:**
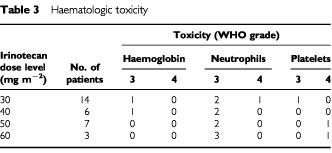
Haematologic toxicity

. Haematologic toxicities were mild. The only grade 4 leukopenia and neutropenia were seen in one patient (grade 4 neutropenia) given 30 mg m^−2^ of irinotecan. G-CSF was administered to five of 14 patients on 30 mg m^−2^ of irinotecan, three of six on 40 mg m^−2^, four of seven on 50 mg m^−2^, and all three on 60 mg m^−2^ dose of irinotecan. Grade 4 thrombocytopenia occurred in two patients (one at the 50 mg m^−2^ and one at 60 mg m^−2^ doses of irinotecan) and this was dose-limiting toxicity. These two patients required platelet transfusions.

### Nonhaematologic toxicity

The nonhaematologic toxicities are summarised in Table 4

**Table 4:**
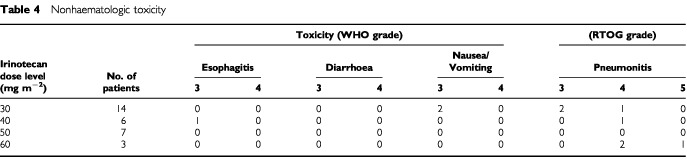
Nonhaematologic toxicity

. One patient suffered from grade 3 esophagitis at an irinotecan dose of 40 mg m^−2^, and two patients had grade 3 nausea with vomiting at 30 mg m^−2^ of irinotecan. No patient suffered from either grade 3 or 4 diarrhea. Grade 4 pneumonitis was observed in two patients treated with 60 mg m^−2^ of irinotecan, as well as in one patient each at both 30 mg m^−2^ and 40 mg m^−2^. Grade 5 pneumonitis was observed in one patient with 60 mg m^−2^ of irinotecan. Grade 4–5 pneumonitis was dose-limiting toxicity and was observed in all three patients at the 60 mg m^−2^ of irinotecan dose. Therefore we decided that this dose was defined as the maximum tolerated dose. Of these five patients who had grade 4–5 pneumonitis, all were treated with steroids and three required mechanical ventilation. Four patients eventually recovered, however one patient given 60 mg m^−2^ of irinotecan suffered treatment-related death. Pneumonitis seemed to be a principal toxicity of this combined modality.

### Response

The response to treatment is summarised in Table 5

**Table 5:**
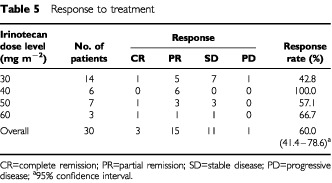
Response to treatment

. Three patients achieved complete remission and 15 patients achieved partial remission, for an overall objective response rate of 60.0% (95% confidence interval 41.4–78.6%). Among the 18 responders, five patients underwent surgical resection of their residual disease and five received systemic chemotherapy with cisplatin and vindesine. Among the 11 patients with stable disease, four also received systemic chemotherapy.

### Survival and duration of response

**Figure 1:**
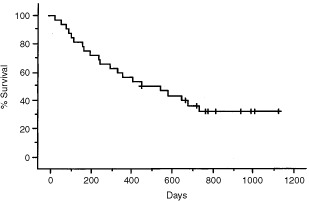
Overall survival. The estimated 1- and 2-year survival rate were 51.6 and 34.2%, and the median survival time was 14.9 months.

The overall median survival time (MST) was 14.9 months, while the 1-year and 2-year survival rates were 51.6% and 34.2%, respectively. In the responding patients (i.e., those who achieved either complete or partial remission), the median duration of response was 11.0 months. In the patients who had either surgery or adjuvant chemotherapy, the MST was 21.9 months (range: 7.8 to 33.0 months) and 24.3 months (range: 5.4 to 32.4 months), respectively. In the other patients, the MST was 10.6 months (range: 1.1 to 36.9 months). The overall survival of all the patients is plotted in Figure 1

.

### Pattern of failure

The sites of initial relapse are shown in Table 6

**Table 6:**
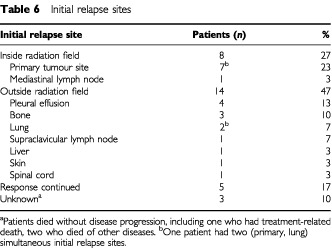
Initial relapse sites

. There were 22 sites of relapse in 29 patients who had partial remission or stable disease. The primary tumour inside the radiation field was the site of initial relapse in eight patients (seven without and one with distant metastasis), while distant metastasis was in ten patients and pleural effusion in four patients. Of five patients who underwent surgery, three patients had no relapse, one died of another disease, and one had pulmonary metastasis.

## DISCUSSION

Our present study showed that the combination of daily low-dose carboplatin and weekly irinotecan with concurrent thoracic radiotherapy is feasible. All three patients who received 60 mg m^−2^ of irinotecan developed grade 4–5 pneumonitis, although grade 4–5 pneumonitis was not observed at the 50 mg m^−2^ dose. In our former study of a phase I/II study (Takeda *et al*, 1999) of weekly irinotecan alone and concurrent thoracic radiotherapy in patients with stage III NSCLC, radiation therapy (2 Gy daily to a total dose of 60 Gy) was performed concurrently with administration of irinotecan done once weekly for 6 weeks. Twenty-seven patients were enrolled at three irinotecan dose levels (30, 45 and 60 mg m^−2^). In that phase I study, grade 4 pneumonitis occurred in one patient at a dose of 60 mg m^−2^, while in the phase II study using 45 mg m^−2^, one out of 10 patients developed severe toxicity (grade 4 pneumonitis plus grade 3 diarrhea) and died. In our study, the irinotecan administration period was reduced from 6 to 4 weeks because in our former study (Takeda *et al*, 1999) the number of patients increased who experienced the skip of the 5th and/or 6th administration of irinotecan. On the former study we added the daily carboplatin as another radiosensitiser.

Development of pulmonary toxicity is generally thought to be related to radiation dose, method of fractionation, and volume of the lung irradiated (Ginsberg *et al*, 1993). In patients receiving combined chemoradiotherapy, other confounding factors, such as the type of chemotherapeutic agent, also may play an important role in determining the risk of this toxicity. New chemotherapeutic agents, such as paclitaxel, have also been reported to show pulmonary toxicity (Choy *et al*, 1998). Therefore, the mechanism of pneumonitis seemed to be an interaction between all three parts of the treatment.

Recent studies suggest that analysis of the three-dimensional dose distribution gives useful data for the prediction of pulmonary toxicity (Martel *et al*, 1994; Marks *et al*, 1997; Graham, 1997). We could not calculate radiotherapy volume data since three-dimensional (3D) radiation therapy were not available with our study. So we calculated radiation portal size by two-dimensional treatment planning data. Radiation portal size was range from 105 m^2^ to 322 m^2^ (mean±SD; 179.7±48.0 m^2^). For five patients with grade 4 or 5 pulmonary toxicity, radiation field size was range from 168 m^2^ to 304 m^2^ (mean±SD; 208.8±54.4 m^2^). There was no significant relationship between radiation field size and pulmonary toxicity. It is very difficult to interpret the toxicity without more information about radiation volume data. This study thinks it is also worth reporting the premorbid lung function data, so we collected the individual data of pulmonary function tests (PFTs) before radiotherapy. Premorbid lung function data (including spirometry, volume measurements, and diffusion capacity) as follows (mean±SD): the per cent predicted vital capacity (%VC) 89.4±19.2%; the forced expiratory volume in 1 sec (FEV1) 1.88±0.58 L; the per cent predicted diffusion capacity to carbon monoxide (%DLCO) 90.1±21.7%. For five patients with grade 4 or 5 pulmonary toxicity, lung function data as follows (mean±SD): %VC 93.6±15.9%; FEV1.0 1.91±0.67L; %DLCO 78.0±23.5%. There was no relationship between PFT parameters and pulmonary toxicities. According these limited information, we suggest that pulmonary toxicity may be drug related rather than field size or baseline PFTs. In our study, radiation volume was not estimated, so we have to plan further study to reveal whether a dose and radiation volume are related to the occurrence of pulmonary toxicity.

The efficacy of combined-modality therapy for inoperable stage III NSCLC is reported to vary and the reason for this is unclear, although differences between the eligibility criteria used in various studies may account for the different outcomes (Mattson *et al*, 1988; Morton *et al*, 1991; Sause *et al*, 1995). In the present study, the maximum tolerated dose of irinotecan was 60 mg m^−2^. Pneumonitis, esophagitis, thrombocytopenia and neutropenia were the dose-limiting toxicities and pneumonitis was the principal toxicity of this regimen, whereas myelosuppression was mild. The overall response rate was 60.0%, while in patients given 50 and 40 mg m^−2^ of irinotecan, the response rate was 57.1 and 100%, respectively. At an irinotecan dose of 40 or 50 mg m^−2^, pneumonitis was manageable and haematologic toxicity was mild. Based on our findings we therefore decided that 50 mg m^−2^ of irinotecan was the recommended dose. Although these data are still preliminary, the median survival time was 14.9 months and the 1- and 2-year survival rates were 51.6 and 34.2%, respectively, which were reasonably good. When comparing these results with similar combined modality studies, the MST and survival rates are most encouraging. And the MST of 21.9 months and 24.3 months in patients who underwent surgery or had adjuvant chemotherapy was better than that in patients who had no additional treatment. It is because that optional treatment was done in responding patients or stable patients. It should be discussed whether in responding patients additional treatment is necessary.

In conclusion, irinotecan combined with daily carboplatin for 4 weeks and concurrent thoracic radiotherapy appears to be feasible and improve the survival of patients with unresectable locally advanced NSCLC. MTD and recommended dose of irinotecan were 60 mg m^−2^ and 50 mg m^−2^, respectively. Principal toxicity of this combined modality was pneumonitis. A phase II study of this combination is warranted.

## References

[bib1] American Society of Clinical Oncology1997 Clinical practice guidelines for the treatment of unresectable non- small-cell lung cancer. Adopted on May 16, 1997 J Clin Oncol 15 29963018 9256144 10.1200/JCO.1997.15.8.2996

[bib2] AtagiSKawaharaMOgawaraMMatsuiKMasudaNKudohSNegoroSFuruseK2000 Phase II trial of daily low-dose carboplatin and thoracic radiotherapy in elderly patients with locally advanced non-small cell lung cancer Jpn J Clin Oncol 30 5964 10768867 10.1093/jjco/hyd022

[bib3] ChoyHAkerleyWSafranHClarkJRegeVPapaAGlantzMPuthawalaYSoderbergCLeoneL1994 Phase I trial of outpatient weekly paclitaxel and concurrent radiation therapy for advanced non-small cell lung cancer J Clin Oncol 12 26822686 7989944 10.1200/JCO.1994.12.12.2682

[bib4] ChoyHSafranHAkerleyWGrazianoSLBogartJAColeBF1998 Phase II trial of weekly paclitaxel and concurrent radiation therapy for locally advanced non-small cell lung cancer Clin Cancer Res 4 19311936 9717821

[bib5] DillmanROSeagrenSLPropertKJGuerraJEatonWLPerryMCCareyRWFreiIIIEFGreenMR1990 A randomized trial of induction chemotherapy plus high-dose radiation versus radiation alone in stage III non-small-cell lung cancer N Engl J Med 323 940945 2169587 10.1056/NEJM199010043231403

[bib6] DoupleEBRichmondRCO'HaraJACoughlinCT1985 Carboplatin as potentiator of radiation therapy Cancer Treat Rev 12 111114 3910216 10.1016/0305-7372(85)90026-x

[bib7] FukuokaMNiitaniHSuzukiAMotomiyaMHasegawaKNishiwakiYKuriyamaTAriyoshiYNegoroSMasudaN1992 A phase II study of CPT-11, a new derivative of camptothecin, for previously untreated non-small-cell lung cancer J Clin Oncol 10 1620 1309380 10.1200/JCO.1992.10.1.16

[bib8] FukuokaMNagaoKOhashiYNiitaniH2000 Impact of irinotecan (CPT-11) and cisplatin (CDDP) on survival in previously untreated metastatic non-small cell lung cancer (NSCLC) Proc Am Soc Clin Oncol 19 495a

[bib9] GinsbergRJKrisMGArmstrongJG1993 Cancer of the lung: Non-small cell lung cancerInCancer: Principles and Practice of Oncology4th edn.,DeVita VT, Hellman S, Rosenberg S (eds)pp676723Philadelphia: Lippincott

[bib10] GrahamMV1997 Predicting radiation response Int J Radiat Oncol Biol Phys 39 561562 9336132 10.1016/s0360-3016(97)00353-2

[bib11] GrecoFAStroupSLGrayJRHainsworthJD1996 Paclitaxel in combination chemotherapy with radiotherapy in patients with unresectable stage III non-small cell lung cancer J Clin Oncol 14 16421648 8622083 10.1200/JCO.1996.14.5.1642

[bib12] GregorA1997 Gemcitabine plus radiotherapy for non-small cell lung cancer Semin Oncol 24 3941 9207316

[bib13] GroenHJvan der LeestAHde VriesEG1995 Continuous carboplatin infusion during 6 weeks' radiotherapy in locally inoperable non-small-cell lung cancer Br J Cancer 72 992997 7547255 10.1038/bjc.1995.448 PMC2034056

[bib14] HerscherLLHahnSMKroogGPassHTemeckBGoldspielBCookJMitchellJBLiebmannJ1998 Phase I study of paclitaxel as radiation sensitizer in the treatment of mesothelioma and non-small-cell lung cancer J Clin Oncol 16 635641 9469352 10.1200/JCO.1998.16.2.635

[bib15] JeremicBShibamotoYAcimovicLDjuricL1995 Randomized trial of hyperfractionated radiation therapy with or without concurrent chemotherapy for stage III non-small-cell lung cancer J Clin Oncol 13 452458 7844608 10.1200/JCO.1995.13.2.452

[bib16] JeremicBShibamotoYAcimovicLMilisavljevicS1996 Hyperfractionated radiation therapy with or without concurrent low-dose daily carboplatin/etoposide for stage III non-small-cell lung cancer: a randomized study J Clin Oncol 14 10651070 8648358 10.1200/JCO.1996.14.4.1065

[bib17] KaplanELMeierP1958 Nonparametric estimation from incomplete observations J Am Stat Assoc 53 457481

[bib18] KunitohHWatanabeKNagatomoAOkamotoHKimbaraK1997 Concurrent daily carboplatin and accelerated hyperfractionated thoracic radiotherapy in locally advanced non-small cell lung cancer Int J Radiat Oncol Biol Phys 1 103109 10.1016/s0360-3016(96)00474-9 9054883

[bib19] Le ChevalierTArriagadaRQuoixERuffiePMartinMTarayreMLacombe-TerrierMJDouillardJYLaplancheA1991 Randomized trials of radiotherapy alone versus combined chemotherapy and radiotherapy in unresectable non-small cell lung cancer: first analysis of a randomized trial in 353 patients J Natl Cancer Inst 83 417423 1847977 10.1093/jnci/83.6.417

[bib20] LeonardCEChanDCChouTCKumarRBunnPA1996 Paclitaxel enhances *in vitro* radiosensitivity of squamous carcinoma cell lines of the head and neck Cancer Res 56 51985204 8912857

[bib21] MarksLBMunleyMTBentelGCZhouSMHollisDScarfoneCSibleyGSKongFMJirtleRJaszczakRColemanRETapsonVAnscherM1997 Physical and biological predictors of changes in whole-lung function following thoracic irradiation Int J Radiat Oncol Biol Phys 39 563570 9336133 10.1016/s0360-3016(97)00343-x

[bib22] MartelMKTen HakenRKHazukaMBTurrisiATFraassBALichterAS1994 Dose-volume histogram and 3-D treatment planning evaluation of patients with pneumonitis Int J Radiat Oncol Biol Phys 28 575581 8113100 10.1016/0360-3016(94)90181-3

[bib23] MattsonKHolstiLRHolstiPJakobssonMKajantiMLiippoKMantylaMNiitamo-KorhonenSNikkanenVNordmanE1988 Inoperable non-small-cell lung cancer: radiation with or without chemotherapy Eur J Cancer Clin Oncol 24 477482 2838288 10.1016/s0277-5379(98)90020-7

[bib24] MauersAMMastersGHarafDJHoffmanPCWatsonSMGolombHMVokesEE1998 Phase I study of docetaxel with concomitant thoracic radiation therapy J Clin Oncol 16 159164 9440738 10.1200/JCO.1998.16.1.159

[bib25] McGinnCJSherwachDSLawrenceTS1996 Radiosensitizing nucleosides J Natl Cancer Inst 88 11931203 8780628 10.1093/jnci/88.17.1193

[bib26] MortonRFJettJRMcGinnisWLEafleJDTherneauTMKrookJEElliottTEMailliardJANelimarkRAMaksymiukAW1991 Thoracic radiation therapy alone compared with combined chemoradiotherapy for locally unresectable non-small cell lung cancer: a randomized, phase III trial Ann Intern Med 115 681686 1656827 10.7326/0003-4819-115-9-681

[bib27] OkishioKKudohSKuriharaNHirataKYoshikawaJ1996 Irinotecan (CPT-11) enhances the radiosensitivity of lung cancer cells *in vitro* Cell Pharmacol 3 247252

[bib28] RobertFChildsHASpencerSAReddenDTHawkinsMM1999 Phase I/IIa Study of Concurrent Paclitaxel and Cisplatin With Radiation Therapy in Locally Advanced Non-Small Cell Lung Cancer: Analysis of Early and Late Pulmonary Morbidity Semin Radiat Oncol 2Suppl 1136147 10210553

[bib29] SauseWTScottCTaylorSJohnsonDLivingstonRKomakiREmamiBCurranWJByhardtRWTurrisiAT1995 Radiation Therapy Oncology Group (RTOG) 88-08 and Eastern Cooperative Oncology Group (ECOG) 4558: preliminary results of a phase III trial in regionally advanced, unresectable non-small-cell lung cancer J Natl Cancer Inst 87 198205 7707407 10.1093/jnci/87.3.198

[bib30] Schaake-KoningCvan den VogaertWDalesioOFestenJHoogenhoutJvan HouttePKirkpatrickAKoolenMMaatBNijsA1992 Effects of concomitant cisplatin and radiotherapy on inoperable non-small-cell lung cancer N Engl J Med 326 524530 1310160 10.1056/NEJM199202203260805

[bib31] TakedaKNegoroSKudohSOkishioKMasudaNTakadaMTanakaMNakajimaTTadaTFukuokaM1999 Phase I/II study of weekly irinotecan and concurrent radiation therapy for locally advanced non-small cell lung cancer Br J Cancer 79 14621467 10188891 10.1038/sj.bjc.6690233 PMC2362741

[bib32] TishlerRBGeardCRHallEJSchiffPB1992 Taxol sensitizes human astrocytoma cells to radiation Cancer Res 52 34953497 1350755

[bib33] World Health Organization1979 WHO Handbook for Reporting Results of Cancer TreatmentWHO Publication No.48. Geneva, Switzerland: World Health Organization

